# *Leptolyngbya* sp. NIVA-CYA 255, a Promising Candidate for Poly(3-hydroxybutyrate) Production under Mixotrophic Deficiency Conditions

**DOI:** 10.3390/biom12040504

**Published:** 2022-03-26

**Authors:** Alexander Kettner, Matthias Noll, Carola Griehl

**Affiliations:** 1 Competence Center Algal Biotechnology, Department of Applied Biosciences and Process Engineering, Anhalt University of Applied Sciences, Bernburger Strasse 55, 06366 Koethen, Germany; alexander.kettner@hs-anhalt.de; 2 Institute of Bioanalysis, Coburg University of Applied Sciences and Arts, Friedrich-Streib-Str. 2, 96450 Coburg, Germany; matthias.noll@hs-coburg.de

**Keywords:** cyanobacteria, fluorescence, FTIR, glycogen, *Leptolyngbya*, LipidGreen2, Nile red, PHB

## Abstract

Cyanobacteria are a promising source for the sustainable production of biodegradable bioplastics such as poly(3-hydroxybutyrate) (PHB). The auto-phototrophic biomass formation is based on light and CO_2_, which is an advantage compared to heterotrophic PHB-producing systems. So far, only a handful of cyanobacterial species suitable for the high-yield synthesis of PHB have been reported. In the present study, the PHB formation, biomass, and elemental composition of *Leptolyngbya* sp. NIVA-CYA 255 were investigated. Therefore, a three-stage cultivation process was applied, consisting of a growth stage; an N-, P-, and NP-depleted phototrophic stage; and a subsequent mixotrophic deficiency stage, initiated by sodium acetate supplementation. The extracted cyanobacterial PHB was confirmed by FTIR- and GC-MS analyses. Furthermore, the fluorescent dyes LipidGreen2 and Nile red were used for fluorescence-based monitoring and the visualization of PHB. LipidGreen2 was well suited for PHB quantification, while the application of Nile red was limited by fluorescence emission crosstalk with phycocyanin. The highest PHB yields were detected in NP- (325 mg g^−1^) and N-deficiency (213 mg g^−1^). The glycogen pool was reduced in all cultures during mixotrophy, while lipid composition was not affected. The highest glycogen yield was formed under N-deficiency (217 mg g^−1^). Due to the high carbon storage capacity and PHB formation, *Leptolyngbya* sp. NIVA-CYA 255 is a promising candidate for PHB production. Further work will focus on upscaling to a technical scale and monitoring the formation by LipidGreen2-based fluorometry.

## 1. Introduction

Plastics have been an essential part of the modern human lifestyle since their discovery at the beginning of the 20th century. Unfortunately, their de facto non-existent biodegradability is a significant disadvantage [1]. The longevity causes accumulation in the terrestrial and marine ecosphere [2]. According to a recent forecast, 12 billion tons of plastic waste will remain in landfills in the environment by 2050, and approximately 12 billion tons of greenhouse gas carbon dioxide (CO_2_) will be released [3]. Considering this trend, biodegradable and renewable alternatives to conventional plastics are becoming increasingly important. A promising class of bioplastics are polyhydroxyalkanoates (PHAs). PHAs are lipophilic polyesters that serve as C and energy storage compounds [4]. They are subcategorized according to their monomer length into short-chain-length (scl-PHAs), medium-chain-length (mcl-PHAs), and long-chain-length PHAs (lcl-PHAs) [5]. Scl-PHAs include the most abundant and most studied representative poly(3-hydroxybutyrate) (PHB). The formation of PHB has been reported in all domains of life, including *Archaea*, *Bacteria*, and *Eukarya* [6,7,8,9,10]. The material properties of PHB are similar to those of polypropylene, with low oxygen permeability, insolubility in water, and thermoplastic behavior [11,12]. In contrast to polypropylene, PHB is completely degraded to water and CO_2_ under aerobic conditions by extracellular depolymerases, and to methane and CO_2_ under anaerobic conditions [13]. 

Bacterial PHB synthesis is, among other factors, initiated by the depletion of macronutrients (nitrogen, N; phosphorus, P) and a simultaneous oversupply of C source(s). Three enzymatic steps catalyze the formation in the cytosol. Starting from acetyl-coenzyme A (acetyl-CoA), the enzyme β-ketothiolase (PhaA) catalyzes the formation of acetoacetyl-CoA via the condensation of two units of acetyl-CoA. Subsequently, acetoacetyl-CoA is reduced to 3-hydroxybutyryl-CoA by the acetoacetyl-CoA-reductase (PhaB), whereby nicotinamide adenine dinucleotide phosphate (NADPH) serves as the electron donor. In the final step, PHB is formed by polymerization of 3-hydroxybutyryl-CoA catalyzed by PHB synthase/polymerase (PhaC) [14,15]. PHB is produced industrially by the heterotrophic cultivation of bacteria such as *Cupriavidus necator, Bacillus* sp., or recombinant *Escherichia coli* [16]. Therefore, large amounts of C sources, such as glucose, fructose, or starch, are needed for the product and biomass formation in combination with mineral salts. The cultivation accounts for approximately 50% of the production costs [17,18]. The high production costs are the primary drawback of widespread use [19]. Therefore, further improvements such as continuous cultivation, the use of wastewater as a nutrient source, and optimization of the product recovery are necessary to bring PHA into a competitive position with petroleum-based plastics [20,21]. Another alternative approach is the cultivation of photoautotrophic microorganisms such as cyanobacteria as production hosts [22]. The ability of cyanobacteria to synthesize PHB was demonstrated in 1966 [23]. Cyanobacteria uses oxygenic photosynthesis, which employ atmospheric CO_2_ and light as energy source [24]. Therefore, supplementation with an organic C source can be omitted, and biomass production becomes more cost-effective [25].

However, compared to heterotrophically grown bacteria, which contain up to 80 wt% PHB, cyanobacteria produce much lower levels, preventing their economically feasible large-scale application [26]. Recent findings have revealed that PHB in cyanobacteria is mainly built up from glycogen, another C storage form synthesized during nutrient-deficient growth [27,28,29]. Hence, low levels of PHB accumulation have been reported under phototrophic-balanced conditions [30,31]. To overcome these limitations, a multi-stage production process was proposed, where (i) biomass was produced, (ii) stress was induced by N-depleted cultivation, and (iii) PHB formation was promoted through mixotrophic conditions [32]. In this way, intracellular PHA content was enhanced up to 48 wt% in *Aulosira fertilissima*, 58 wt% in *Nostoc muscorum* Agardh, and 55 wt% in *Synechococcus* sp. MA19 using either acetate, fructose, or glucose as a substrate [33,34,35]. 

PHB synthesis in cyanobacteria has been reported in different phylogenetic orders [36,37]. Still, little is known about the storage lipid composition in the filamentous genus *Leptolyngbya,* which belongs to the order Synechococcales. *Leptolyngbya* sp. are present in a wide range of habitats, including freshwater, marine, and terrestrial locations, and extreme places, such as thermal springs [38]. Recently, PHB was isolated from *Leptolyngbya valderiana* and characterized by FTIR [39]. Rueda et al. described PHB formation in microbial consortia containing *Leptolyngbya* sp. with 5 wt% PHB [40]. Further studies have demonstrated that *Leptolyngbya* possesses a balanced ratio of intracellular composition and high lipid content, and is therefore a suitable host for potential large-scale applications [41,42]. 

In this study, PHB formation in *Leptolyngbya* sp. NIVA-CYA 255, isolated from egyptian soil, was investigated for the first time [43,44,45]. For this purpose, a three-stage cultivation process was carried out, consisting of an initial biomass growth phase and a two-stage product formation phase. 

## 2. Materials and Methods

### 2.1. Chemicals

Chemicals were purchased in analytical grade either from Carl Roth (Karlsruhe, Germany) or Sigma-Aldrich/Merck (Darmstadt, Germany) unless otherwise specified.

### 2.2. Strain and Cultivation Conditions

*Leptolyngbya* sp. NIVA-CYA 255 was purchased from the Norwegian Culture Collection of Algae (NORCCA) [45]. The cyanobacteria were cultured in 2 L bubble columns. First, biomass was produced in the BG_11_ growth medium for 14 d. The medium consisted of 1.5 g L^−1^ NaNO_3_, 0.04 g L^−1^ K_2_HPO_4_, 0.075 g L^−1^ MgSO_4_ × 7 H_2_O, 0.036 g L^−1^ CaCl_2_ × 2 H_2_O, 0.006 g L^−1^ citric acid, 0.006 g L^−1^ ferric ammonium citrate, 0.001 g L^−1^ Na_2_EDTA × 2 H_2_O, 0.02 g L^−1^ Na_2_CO_3_, 2 g L^−1^ NaCl, and micronutrients: 2.86 µg L^−1^ H_3_BO_3_, 1.81 µg L^−1^ MnCl_2_ × 4 H_2_O, 0.222 µg L^−1^ ZnSO_4_ × 7 H_2_O, 0.39 µg L^−1^ Na_2_MoO_4_ × 2 H_2_O, 0.079 µg L^−1^ CuSO_4_ × 5 H_2_O, 0.0494 µg L^−1^ Co(NO_3_)_2_ × 6 H_2_O based on Rippka et al. [46]. Then, 500 mL inoculum and 1500 mL of N-depleted BG_11_ (BG_11_^N−^), P-depleted (BG_11_^P−^), N- and P-depleted (BG_11_^NP−^), or BG_11_ growth medium as control (BG_11_^C^) was added and incubated for 16 d. Subsequently, 2 g L^−1^ sodium acetate was added, and cultivation continued for an additional 8 d. Each medium was initially adjusted to pH 8.0. Cultivation was performed at 26 °C, illuminated with 100 µmol s^−1^ m^−2^ at a 12 h/12 h light/dark rhythm, and 1 vvm gas flow supplemented with 5% (*v*/*v*) CO_2_. Samples were taken under sterile conditions at regular intervals. 

### 2.3. Biomass Concentration and Elemental Composition

Cyanobacterial growth was followed gravimetrically. Therefore, 6–9 mL of cell culture was centrifuged (Thermo Scientific, Waltham, MA, USA) at 10,000× *g* for 5 min, washed, frozen, and dried by lyophilization (Christ Martin, Osterode, Germany). Biomass concentration was calculated from the ratio of dried biomass to cell culture volume. The elemental composition of the biomass was determined by CHNS composition using a Vario MICRO Cube (Elementar Analysensysteme, Langenselbold, Germany) equipped with a thermal conductivity detector. Protein content was calculated from the N-content as described previously [47].

### 2.4. PHB and Glycogen Content

PHB and glycogen were quantified by HPLC, applying slightly modified conditions as reported elsewhere [48,49,50]. Briefly, 500 µL of 75% H_2_SO_4_ or 500 µL of 7.5% H_2_SO_4_ were added to the retained dried biomass for PHB or glycogen extraction, respectively, and heated at 95 °C for 60 min. The reaction mixture was diluted with distilled water before measurement. Standards were treated alike and used for calibration. Isocratic separation was performed with a Merck-Hitachi HPLC and ABOA SugarSep column (AppliChrom, Oranienburg, Germany) with 0.007 N H_2_SO_4_ as mobile phase and a flow rate of 0.8 mL min^−1^ at 50 bar. *Cis*- and *trans*-crotonic acid, as the hydrolysis products of PHB, were identified and quantified with a UV detector at 214 nm. As the hydrolysis product of glycogen, glucose was detected with a Merck-Hitachi, L-7490 refractive index detector.

### 2.5. PHA Extraction 

PHA was extracted from 10–20 mg dried biomass with 10 mL chloroform in sealed glass extraction tubes for 60 min at 70 °C. Extracts were separated from residual biomass by hot filtration, precipitated with 5 mL cold ethanol, filtered, washed with 20 mL acetone and 40 mL water, and dried at 60 °C overnight.

### 2.6. FTIR-Analysis

Crude extracted PHA was analyzed for conformity with Fourier-transform infrared spectroscopy (FTIR) using a Bruker Tensor 27 (Bruker Corp., Billerica, MA, USA) equipped with an attenuated total reflection unit (ATR). Interferograms were taken between 550 and 4000 cm^−1^. Samples were scanned 30 times at a 4 cm^−1^ resolution. The resulting pattern of functional groups were compared to the PHB standard. 

### 2.7. Monomer Composition

GC-MS was applied for the determination of the PHA composition. Analysis of standard poly(3-hydroxybutyrate-co-3-hydroxyvalerate) (PHBHV) and extracted PHA-polyester was performed using a previously reported method [8]. Briefly, 5 mg polyester was simultaneously transesterified and hydrolyzed in sealed headspace vials using 4 mL 1,2-dichloroethane, 2 mL 4:1 (vol/vol) propanol HCl- mixture, and 100 µL of 20 g L^−1^ benzoic acid as internal standard. Propanolysis was carried out at 120 °C for 4 h. Afterward, 4 mL water was added, mixed, and maintained until phase reseparation. Then, 1000 µL of the lower (organic) phase was transferred for GC-MS analysis. Separation occurred on a Stabilwax column (Restek, Bad Homburg, Germany) using He as carrier at a flow rate of 1.44 mL min^−1^, and a gradient of 120 °C (3 min), 140 °C at 3 °C min^−1^, 230 °C at a heating ramp of 50 °C min^−1^, and 240 °C at 10 °C min^−1^. A standard curve of PHBHV (12 wt% 3HV, Sigma-Aldrich/Merck, Darmstadt, Germany) was used for the qualification of propionyl-3HB and propionyl-3HV monomers. The resulting chromatograms and mass to charge ratio (m/z) were analyzed to identify monomer composition. 

### 2.8. FAME and Lipid Classes

Simultaneous extraction and transesterification for fatty acid methyl ester (FAME) quantification were conducted in sealed headspace vials using 3 mL 3 N methanolic HCl and 4 mL hexane. Subsequently, 3 mL H_2_O was added and homogenized. After phase separation, the upper phase was transferred via a syringe filter into a corresponding vial. 

GC-MS analysis was conducted with He as mobile phase at 48 kPa, 250 °C, and a flow rate of 7.7 mL min^−1^, and SGE BPX 70 column as stationary phase (Fisher Scientific, Schwerte, Germany) using a QP2010Plus (Shimadzu, Kyoto, Japan).

For the determination of lipid class composition, dry biomass was extracted with chloroform and methanol at a ratio of 2:1 (*v*/*v*) from dried biomass using an MM200 bead mill for homogenization (Retsch, Haan, Germany). Subsequently, the extract was separated by centrifugation at 10,000× *g* for 10 min. The chloroform phase was collected, and lipids were concentrated by evaporation of the chloroform (CombiDancer, Hettich, Kirchlengern, Germany). Lipid classes were separated with a LaChrom HPLC (Merck-Hitachi, Kyoto, Japan) coupled to an evaporative Sedex 90 light scattering detector (ELSD) as outlined previously [51]. Briefly, a binary gradient elution at a constant flow rate of 0.8 mL min^−1^, and the eluents toluene:isopropanol:acetic acid:triethylamine (A, 95:5:0.2:0.1), isopropanol:methanol:acetic acid:triethylamine (B, 60:40:0.2:0.1), and methanol:triethylamine (C, 100:0.2) were applied using the gradient 0–10 min 100% A, 10–15 min 50% A 50% B, 15–18 min 50% A 50% C, 18–25 min 10% A 90% C, 25–35 min 100% C. As the stationary phase, a LiChrospher 100 Diol (5 µm) column (Merck, Darmstadt, Germany) was applied at 20 °C and a flow rate of 0.8 mL min^−1^. The ELSD evaporation temperature was set to 60 °C at an N_2_ pressure of 3.5 bar. The lipid classes were identified by comparing retention times to commercial analytical standards (Sigma Aldrich, Darmstadt, Germany) and quantified using calibration curves. The lipid class phospholipids (PL) represent the sum of the lipids monogalactosyldiacylglycerol (MGDG), phosphatidylglycerol (PG), sulfoquinovosyl diacylglycerol (SQDG), digalactosyldiacylglycerol (DGDG), phosphatidic acid (PA), and phosphatidylserine (PS).

### 2.9. Fluorescence PHB Quantification with Nile red and LipidGreen2

The fluorescence staining method was carried out based on our own previous studies [52]. Briefly, 1500 µL PBS buffer (137 mM NaCl, 2.7 mM KCl, 10 mM Na_2_HPO_4_, 1.8 mM KH_2_PO_4_, 5 mM EDTA, and 5 mM Tris, pH 7.5) was mixed with 500 µL cell culture in a standard 1 cm cuvette. After 5 min incubation, the fluorescence emission of the PHB-dye-complex was detected with a Perkin Elmer LS45 (Perkin Elmer, Waltham, MA, USA). Slits were set to 10 nm, and a gain of 700 V was applied. The excitation wavelength was set to 440 nm for LipidGreen2 and 525 nm for Nile red. The fluorescence was recorded by an emission scan over 200 nm, starting at 460 nm and 545 nm for LipidGreen2 and Nile red, respectively. Emission intensities at 510 nm (LipidGreen2) and 610 nm (Nile red) were plotted against HPLC-obtained PHB values.

### 2.10. Bioimaging

To observe morphological changes and visualize cyanobacterial PHB granules, incident light and fluorescence microscopic images were captured with Olympus BX41 (Olympus, Tokyo, Japan) equipped with a XC50 camera (Olympus, Tokyo, Japan). The staining was performed as described in Section 2.9. An excitation filter of 460–490 nm was used, and images were analyzed with the cellSens software package (Olympus) as explained by the manufacturer’s instructions.

### 2.11. Data Analysis and Statistics

Data plotting, analysis, and statistics were performed with OriginPro (OriginLab Co, Northampton, MA, USA). A *t*-test at α = 0.1 was used to determine differences between PHB content obtained by either HPLC or fluorimetry. Fluorescence data were normalized to the corresponding analytical values to obtain a normal distribution. All measurements were performed as independent triplicates.

## 3. Results

### 3.1. Morphology and Growth 

**Figure 1:**
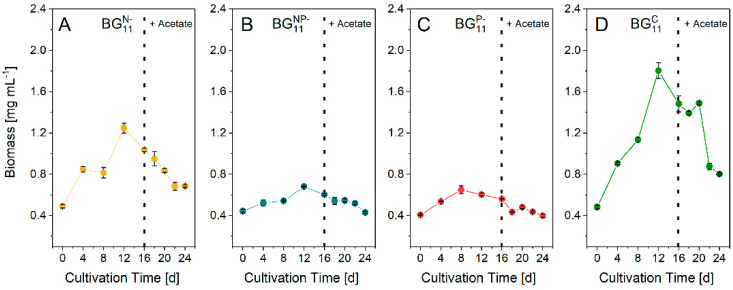
Biomass formation under N-deficiency ((**A**), BG_11_^N−^), N- and P−deficiency ((**B**), BG_11_^NP−^), P-deficiency ((**C**), BG_11_^P−^), and without deficiency ((**D**), control, BG_11_^C^). The dashed vertical line indicates the addition of 2 g L^−1^ sodium acetate and the beginning of the mixotrophic stage. Data are the mean ± SD of three replicates.

Morphological changes between the different deficiency conditions were observed by microscopy at the end of the phototrophic stage (Figure S1). Biomass was monitored during the phototroph-deficient and mixotrophic stage. The highest final cell concentration of 1.48 g L^−1^ and the highest growth rate (64.4 mg L^−1^d^−1^) were reached in BG_11_^C^ at the end of the phototrophic stage. Deficient conditions caused lower productivity (Figure 1). *Leptolyngbya* sp. reacted to the P limitation with meager growth rates of 6.9 mg L^−1^d^−1^ and 9.4 mg L^−1^d^−1^ for BG_11_^P^^−^ and BG_11_^NP^^−^, respectively. N limitation did not affect growth in the same magnitude. However, *Leptolyngbya* sp. cultured in BG_11_^N^^−^ was 44% lower in growth when compared to BG_11_^C^, resulting in 36.3 mg L^−1^d^−1^ and 1.0 g L^−1^ final biomass concentration at the end of the phototrophic stage. Supplementation with sodium acetate (mixotrophic stage) caused a decrement in biomass formation in all cultures. The decrease in BG_11_^P−^ was minimal since the growth rate was already low. The most significant decrease was demonstrated by BG_11_^C^, which lost 48% of biomass during the mixotrophic cultivation phase, resulting in a final biomass concentration of 0.85 g L^−1^ compared to 0.68 g L^−1^ of BG_11_^N−^.

### 3.2. PHB-Formation and Biomass Composition

**Figure 2:**
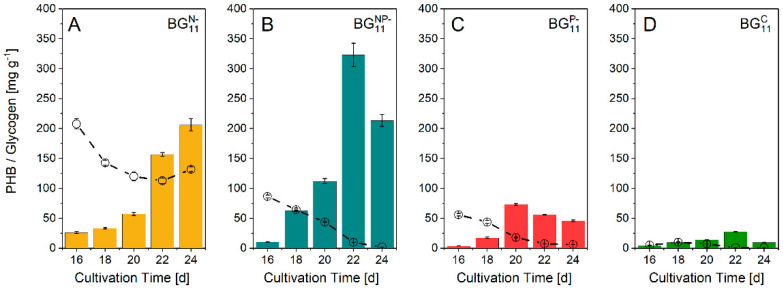
PHB (columns) and glycogen formation (dashed line) under N-deficiency ((**A**), BG_11_^N−^), N- and P-deficiency ((**B**), BG_11_^NP−^), P-deficiency ((**C**), BG_11_^P−^), and without deficiency ((**D**), control, BG_11_^C^), and supplementation with 2 g L^−1^ sodium acetate. Data are the mean ± SD of three replicates.

PHB and glycogen formation were studied during the mixotrophic stage, which was accompanied by a decrease of glycogen in all cultures. The intracellular glycogen was built up in the phototrophic stage and metabolized entirely in BG_11_^P−^ and BG_11_^C^ (Figure 2). Whereas BG_11_^N−^-starved cells showed the highest intracellular content of 217 mg g^−1^ glycogen, BG_11_^NP−^, BG_11_^P−^, and BG_11_^C^ exhibited levels of 87, 58, and 7 mg g^−1^, respectively.

In comparison, cultures grown in BG_11_^N−^ and BG_11_^NP−^ showed a similar glycogen consumption rate of approximately 80 mg g^−1^. However, since the initial glycogen concentration in BG_11_^N−^ was higher than that of BG_11_^NP−^, the glycogen was not completely metabolized until the end of the experiment. 

Low PHB concentrations were present at the beginning of the mixotrophic stage in all cultures. Both BG_11_^P−^ and BG_11_^C^ revealed PHB levels lower than 5 mg g^−1^. After the supplementation with sodium acetate, PHB content increased to a maximum of 74 mg g^−1^ after 4 d, and to 25 mg g^−1^ after 6 d for BG_11_^P−^ and BG_11_^C^, respectively. The highest PHB concentration was observed after 22 d in BG_11_^NP−^. Within 6 d of mixotrophy, the PHB content increased remarkably to 325 mg g^−1^. Subsequently, the concentration decreased to 213 mg g^−1^, as the glycogen concentration decreased. PHB formation in BG_11_^N−^ increased during the mixotrophic stage and reached 206 mg g^−1^ at the end of the experiment, which was comparable to the content of BG_11_^NP−^ (213 mg g^−1^).

In addition to PHB and glycogen monitoring, the final biomass was examined for FAME and lipid group composition, since the presence of triacylglycerides (TAG) was previously described as a storage compound in cyanobacteria [37]. Indeed, all depleted cultures demonstrated a doubling in TAG content compared to the control BG_11_^C^ (Table 1). TAG levels of 13.6 mg g^−1^ (BG_11_^N−^), 15.6 mg g^−1^ (BG_11_^P−^), 16.1 mg g^−1^ (BG_11_^NP−^), and 7.2 mg g^−1^ (BG_11_^P−^) were obtained. Polar lipids (PL) content was lowest in BG_11_^P−^ with 7.4 mg g^−1^. BG_11_^C^, BG_11_^N−^, and BG_11_^NP−^ showed PL levels of 18.4 mg g^−1^, 11.4 mg g^−1^, and 10.3 mg g^−1^, respectively.

C16 and C18 are common fatty acids in neutral lipids such as TAG. The sum of C16 and C18 reached 5.8 mg g^−1^ in the control culture BG_11_^C^. The macronutrient-depleted cultures showed higher levels of 13.2 mg g^−1^ (BG_11_^N−^), 17.9 mg g^−1^ (BG_11_^P−^), and 13.7 mg g^−1^ (BG_11_^NP−^). The lowest protein content was observed in BG_11_^N−^ (13.5 wt%), resulting in a high C/N ratio of 13.0. BG_11_^P−^ (26.1 wt%), and BG_11_^NP−^ (22.0 wt%) demonstrated higher protein contents, which corresponded to half of the protein content of BG_11_^C^ (44.8 wt%). Therefore, the C/N ratios of BG_11_^P−^ and BG_11_^NP−^ were significantly lower than BG_11_^N−^, but only minimally higher than BG_11_^C^. 

### 3.3. Correlation of PHB and Fluorescence

**Figure 3:**
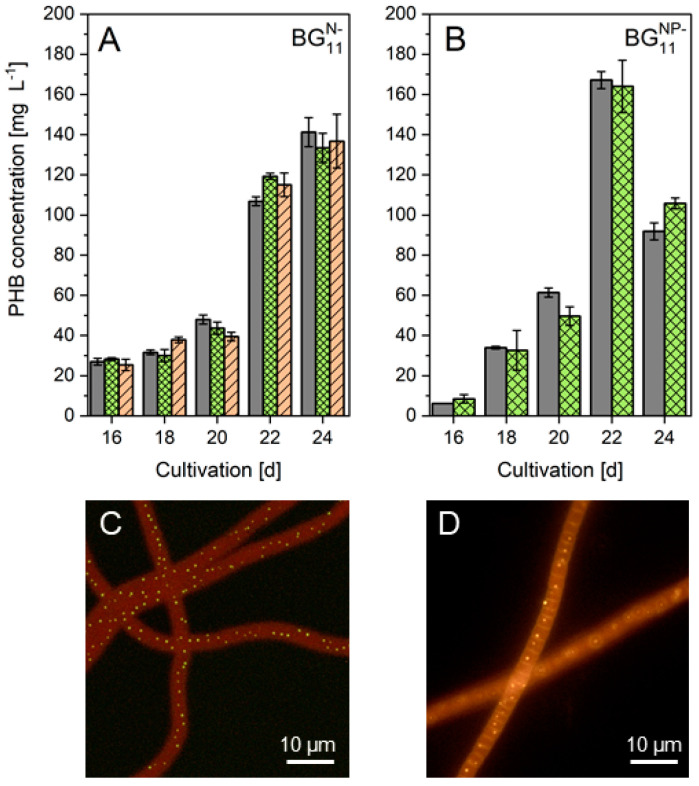
Fluorescent detection and bioimaging of PHB in *Leptolyngbya* sp. NIVA-CYA 255. PHB levels as revealed by HPLC (solid bars, grey), LipidGreen2 (crossed bars, green), and Nile red (dashed bars, orange) of BG_11_^N−^ (**A**) and BG_11_^NP−^ cultures (**B**). Data are the mean ± SD of three replicates. PHB granule bioimaging by LipidGreen2 (**C**) and Nile red (**D**).

The feasibility of fluorescent PHB detection using LipidGreen2 and Nile red staining for quantitative measurements was examined using a correlation of raw fluorescence and HPLC-obtained PHB content in accordance with a previous study [53]. A remarkably high agreement of LipidGreen2 fluorescence to PHB concentration was achieved. The combined coefficient (R^2^) of BG_11_^N−^ and BG_11_^NP−^ was 0.9883 (Figure S2). In contrast, Nile red fluorescence and HPLC-based PHB content were only correlated in the BG_11_^N−^ culture (R^2^ = 0.9484). For BG_11_^NP−^, no linear relationship (R^2^ = 0.3375) was obtained since phycocyanin fluorescence interfered with the fluorescence of Nile red-stained PHB (Figure S3). The regression models were used to calculate and compare the PHB levels (Figure 3A,B). No significant difference was found between HPLC analysis and LipidGreen2 fluorescence [t(18) = 2.1 10^−5^, *p* = 0.999]. The values of all calculated contents using Nile red fluorescence were significantly different than the obtained HPLC contents [t(18) = −1.99, *p* = 0.066]. The fluorescence dyes were further studied for the fluorescence bioimaging of PHB granules (Figure 3C,D). Nile red demonstrated an evident yellow-orange fluorescence emission of PHB granules and cell membrane. Compared to Nile red, LipidGreen2 did not stain the cell membrane.

### 3.4. Characterisation of Extracted Polyester

**Figure 4:**
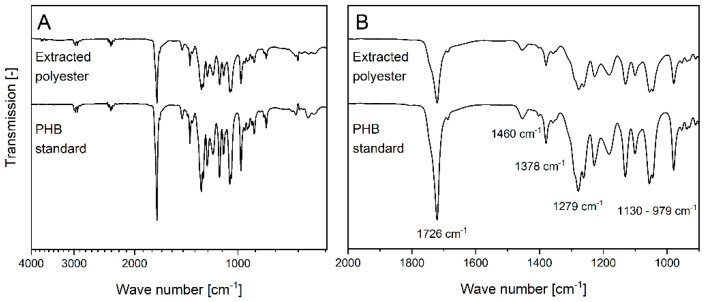
FTIR spectrum of PHB extracted from *Leptolyngbya* sp. NIVA-CYA 255 and PHB standard (**A**). A zoom into wave numbers ranging from 2000 to 800 cm^−1^ (**B**).

The characteristic wave numbers of PHB in the FTIR spectrum were presented by the strong carbonyl group (C=O) at 1726 cm^−1^ and asymmetric C-O-C stretching vibration at 1279 cm^−1^, which are typical for ester bondings in PHB polyesters (Figure 4). The patterns are consistent with earlier reports [54]. Other adsorption bands obtained at 1460 and 1378 cm^−1^ designated the -CH_2_ and -CH_3_ groups, respectively. The fingerprint region from 1130 to 979 cm^−1^ was denoted as the C-O and C-C stretching vibration [31]. With 97.2% agreement, the spectra of the isolated PHB from *Leptolyngbya* sp. NIVA-CYA 255 matched that of the PHB standard.

FTIR spectra do not accurately indicate the exact PHA type, as it is challenging to distinguish between the vibration pattern of different scl-co-polyesters. Therefore, the monomeric composition was analyzed by GC-MS (Figure S4). Compared to the scl-co-polyester standard PHBHV, which constituted two peaks at 3.75 min (hydroxybutyrate, HB) and 4.8 min (hydroxyvalerate, HV), only HB could be detected in the extract of *Leptolyngbya* sp. NIVA-CYA 255. Due to the absence of HV, the formation of homopolymeric PHB could be confirmed. 

## 4. Discussion

Cyanobacteria offer a promising and sustainable alternative to produce biomolecules, such as the polyester PHB. Currently, industrial production of PHB with cyanobacteria is not lucrative due to the production costs and long cultivation time. An initial production analysis assessed the current price of cyanobacterial PHB to be approximately EUR 24 kg^−1^, which is 2–5 times higher than the present synthesis with heterotrophic bacteria [55,56]. To reduce PHB production costs, the cultivation conditions were envisaged to be optimized, and new species was studied. In preliminary experiments, *Leptolyngbya* sp. NIVA-CYA 255 was a suitable candidate for cultivation on a technical scale as the cultivation was robust and not sensitive to temperature fluctuations. Therefore, PHB synthesis and biomass composition were investigated. Studies indicated a significant contribution of PHB to cell dormancy in addition to its function as a storage lipid [57]. It has been demonstrated that PHB is involved in stress survival during environmental changes [58,59,60,61]. PHB is formed from the intracellular glycogen pool built up during the photoautotrophic stage, especially under N-deficiency [62,63]. The results of our study confirmed these findings. The highest glycogen pool was formed during N-deficiency and converted into PHB in the mixotrophic phase. Significantly lower glycogen concentrations were achieved in BG_11_^P−^ and BG_11_^C^ (Figure 2). These results are consistent with earlier reports, where N-deficiency led to a higher total amount of C storage compounds (PHB, glycogen, lipids) in *Synechocystis* sp. PCC 6714 [64].

Consequently, a lack of P during the phototrophic stage led to a decreased glycogen productivity and thus reduced the formation of PHB. P-deficiency also had a substantial negative impact on growth, which was almost completely stopped in the P-deficient cultures BG_11_^NP−^ and BG_11_^P−^ (Figure 1). Nevertheless, the highest PHB content was obtained in BG_11_^NP−^ (Figure 2B). Acetate accelerated the PHB formation and ensured PHB increment within a short time. Although the PHB content of 20.6 wt% in N-deficient culture was lower than that of the NP-deficient culture (32.3 wt%), the higher biomass concentration resulted in a comparable PHB concentration of 141.5 mg L^−1^ compared to 167.3 mg L^−1^ (NP-deficiency). Therefore, N-limitation is beneficial for large-scale applications, since (i) biomass productivity does not stop during the N-deficient stage, (ii) high glycogen productivity (Figure 2) was observed, and (iii) the dormant state (chlorosis) supports PHB production and cell survival. A prolonged cultivation period might result in even higher yields. High PHB concentrations were obtained in similar studies (Table 2).

*Synechococcus* sp. MA19 showed the highest content of 55 wt% under P-deficient conditions (Table 2). However, *Synechococcus* sp. MA19 is a thermophile, resulting in high cultivation temperatures of 50 °C. Due to the additional energy input, higher production costs have to be considered. Considering this fact, *Leptolyngbya* sp. mesophilic cultivation temperatures of 26 °C is advantageous for large-scale applications.

Although PHB is described as a typical storage lipid in cyanobacteria, the formation of lipid bodies has also been reported [62]. Therefore, lipid classes, including TAG as the main component of lipid bodies, were analyzed. Due to the low intracellular content (1.3–1.6 wt%), TAG’s function can be understood as a general stress response rather than a storage compound. 

The highest C/N ratio was obtained with BG_11_^N−^. This value represents a cumulative PHB and glycogen content parameter, and can be considered as a general C storage formation benchmark. However, C/N analysis must be performed from the dried biomass, which is time-consuming and not applicable for rapid monitoring approaches. Therefore, the eligibility of fluorometric monitoring was also studied. Unlike heterotrophic bacteria, cyanobacteria are subjected to major morphological changes under different stress conditions, including the formation or degradation of pigments. For fluorometric detection, interferences of pigment fluorescence should be considered. Nile red PHB emission interfered with phycobilin fluorescence in BG_11_^NP−^ and therefore showed no correlation (Figure S2). 

LipidGreen2 revealed better applicability for PHB detection since the fluorescence emission maximum of 510 nm was outside the phycobilin and chlorophyll fluorescence emission. This resulted in high degrees of correlations for both BG_11_^N−^ and BG_11_^NP−^ cultures. To the best of our knowledge, this is the first report using cuvette-based fluorimetry coupled with LipidGreen2 for PHB monitoring in cyanobacteria. Due to the advantageous properties of the dye, future studies will focus on the process optimization and up-scaling of *Leptolyngbya* NIVA-CYA 255 using LipidGreen2 for the fast monitoring of intracellular PHB contents.

## 5. Conclusions

*Leptolyngbya* sp. NIVA-CYA 255 is a promising host for cyanobacterial PHB production. This study investigated PHB formation in a three-stage cultivation process, containing a growing stage, a macronutrient-depleted phototrophic stage, and a subsequent mixotrophic stage. Cultivation in N- and P-deficiency supplemented with acetate resulted in an intracellular concentration of 32.3 wt% PHB. At the end of the experiment, BG_11_^NP−^ and BG_11_^N−^ cultures demonstrated comparable PHB concentrations. Since BG_11_^N−^ showed the highest C storage capacities (PHB and glycogen), N-depletion seems to be the favorite strategy for PHB production in *Leptolyngbya* sp. NIVA-CYA 255 that can be easily monitored using LipidGreen2-fluorescence.

## Figures and Tables

**Table 1:** Biomass composition of *Leptolyngbya* sp. NIVA-CYA 255 at the end of the mixotrophic stage. FAME: fatty acid methyl ester, PL: sum of polar lipids, TAG: triacylglycerols.

Category	Class	BG_11_^C^	BG_11_^N−^	BG_11_^P−^	BG_11_^NP−^
Biomass and Storage Compounds	Biomass [ mg L^−1^]	841.7	686.7	356.5	371.7
	PHB [mg g^−1^]	9.6	206.0	4.6	213.3
	Glycogen [mg g^−1^]	1.0	132.0	0.6	2.0
Lipid Classes	TAG [mg g^−1^]	7.2	13.6	15.6	16.1
	PL [mg g^−1^]	18.4	11.4	7.4	10.3
FAME	C16 [mg g^1^]	5.8	10.1	9.2	7.3
	C18 [mg g^−1^]	-	3.1	8.7	5.4
Elementary Analysis	Protein [wt%]	44.8	13.5	26.1	22.0
	C/N ratio	4.5	13.0	5.5	8.6

**Table 2:** Production of PHB by cyanobacteria under conditions similar those in this study.

Cyanobacteria	Deficiency	Supplement	Biomass [mg L^−1^]	PHB [wt%]	Reference
*Leptolyngbya* sp. NIVA-CYA 255	N, P	Acetate	517.8	32.3	This study
	N	Acetate	686.7	20.6	This study
	P	Acetate	481.8	7.4	This study
*Synechocystis* sp. PCC6803	P	Acetate, Glucose	110.0	29.0	Panda et al. [65]
*Nostoc muscorum* Agadh	N	Acetate	465.4	28.0	Bhati et al. [33]
*Aulosira fertilissima* CCC 444	N	Acetate	631	48.7	Samantaray et al. [35]
*Synechococcus* sp. MA19	P		4400	55.0	Nishioka et al. [34]
*Spirulina* sp. LEB18	N	Biopolymer waste	500	30.7	Coelho et al. [66]
*Synechocystis* sp. PCC 6714	N, P	Acetate	1900	16.4	Kamravamanesh et al. [67]

## Data Availability

Datasets were generated and analyzed during the study.

## Supplementary Materials

The following are available online at https://www.mdpi.com/article/10.3390/biom12040504/s1, Figure S1: Morphological changes of *Leptolyngbya* sp. NIVA-CYA 255, Figure S2: Linear agreement of LipidGreen2 and Nile red fluorescence, Figure S3: Fluorescence emission signal overlap of phycocyanin and Nile red during PHB staining, Figure S4: GC-MS chromatograms and mass spectra of PHBHV standard and PHB extracted from *Leptolyngbya* sp. NIVA-CYA 255.
