# Supplementary material for: Leptolyngbya sp. NIVA-CYA 255, a Promising Candidate for Poly(3-hydroxybutyrate) Production under Mixotrophic Deficiency Conditions
Source: Biomolecules. 2022 Mar 26;12(4):504. doi: 10.3390/biom12040504 (PMC9030801; doi:10.3390/biom12040504)
Supplement: Supplementary file 1 [file biomolecules-12-00504-s001.zip › biomolecules-1648640-supplementary.pdf]

Supplementary Figures

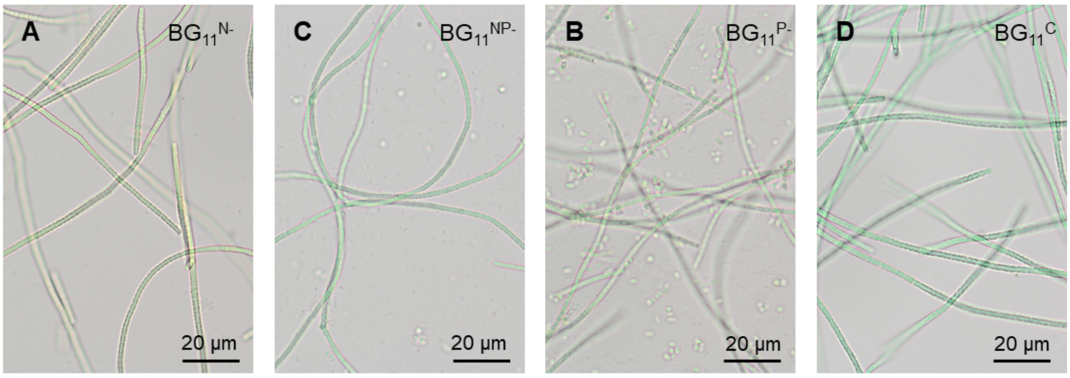

Figure S1. Morphological changes of *Leptolyngbya* sp. NIVA-CYA 255 during nitrate-deficiency, (BG_{11}^{N-}, **A**), nitrate- and phosphate- deficiency, (BG_{11}^{NP-}, **B**), phosphate- deficiency, (BG_{11}^{P-}, **C**), compared to the control culture without any deficiency, (BG_{11}^{C}, **D**). Images were taken after 14 d of cultivation.

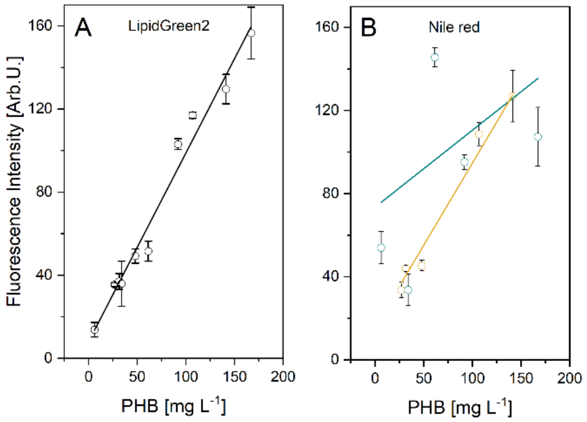

Figure S2. Linear agreement (R^2 = 0.9883) of LipidGreen2 fluorescence of combinded BG_{11}^{N-} and BG_{11}^{NP-} cultures **(A)** and agreements of Nile red fluorescence **(B)** of BG_{11}^{N-} (Orange, R^2 = 0.9484) or BG_{11}^{NP-} (Blue, R^2 = 0.3375) to analytical PHB values.

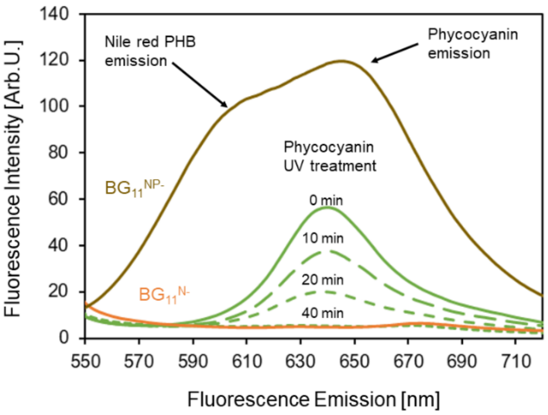

Figure S3. Fluorescence emission signal overlap of phycocyanin and Nile red during PHB staining of *Leptolyngbya* sp. NIVA-CYA 255. The presence of phycocyanin (emission peaks highlighted in green) can be eliminated by UV_{234} radiation for at least 40 min. After UV treatment, phycocyanin emission was comparable to

BG$_{11}$$^{N-}$, which did not show incident phycocyanin fluorescence (orange). Emission spectra were obtained at 525 nm excitation.

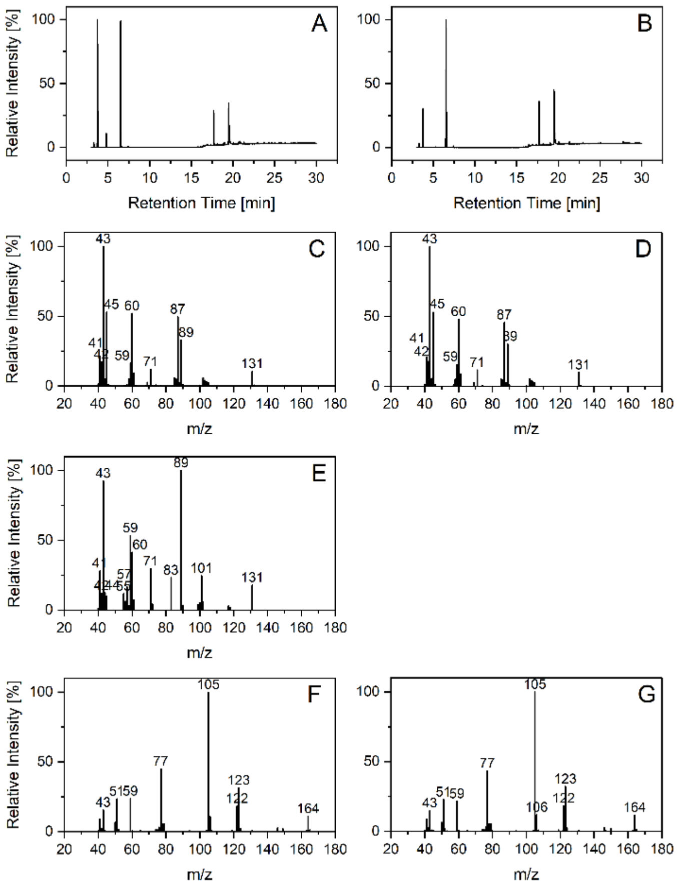

Figure S4. GC-MS chromatograms and mass spectra of PHBHV standard [left row] and PHB extracted from *Leptolyngbya* sp. NIVA-CYA 255 [right row]. Retention times: 3.75 min: hydroxybutyrate **(C, D)**, 4.8 min: hydroxyvalerate **(E)**, 6.5 min: benzoic acid, **(F, G)**.
